# Antiviral activity of *Bifidobacterium adolescentis* SPM1605 against Coxsackievirus B3

**DOI:** 10.1080/13102818.2014.945237

**Published:** 2014-10-20

**Authors:** Min Ji Kim, Do Kyung Lee, Jae Eun Park, Il Ho Park, Jae Gu Seo, Nam Joo Ha

**Affiliations:** ^a^ College of Pharmacy, Sahmyook University, Seoul, Republic of Korea; ^b^ R&D Center, Cellbiotech, Co. Ltd., Gimpo, Republic of Korea

**Keywords:** Coxsackievirus, *Bifidobacterium adolescentis*, HeLa cells, plaque reduction assay, real-time qPCR

## Abstract

Bifidobacteria are considered one of the most beneficial probiotics and have been widely studied for their effects against specific pathogens. The present study investigated the antiviral activity of probiotics isolated from Koreans against Coxsackievirus B3 (CVB3). The effect of probiotic isolates against CVB3 was measured by the plaque assay and cellular toxicity of bifidobacteria in HeLa cells was measured using 3-(4,5-dimethylthiazol-2-yl)-2,5-diphenyltetrazolium bromide (MTT) assay. Among 13 probiotic isolates, 3 *Bifidobacterium adolescentis*, 2 *Bifidobacterium longum* and 1 *Bifidobacterium pseudocatenulatum* had an antiviral effect against CVB3, while the others did not show such effect. *B. adolescentis* SPM1605 showed the greatest inhibitory properties against CVB3. When the threshold cycle (CT) values for the treated *B. adolescentis* SPM1605 samples were compared to the results for the non-treated samples, it was shown that the amplified viral sequences from the CVB3 had their copy number lowered by *B. adolescentis* SPM1605. Moreover, the gene expression in infected HeLa cells was also inhibited by 50%. The results suggest that *B. adolescentis* SPM1605 suppresses CVB3 and could be used as an alternative therapy against infectious diseases caused by coxsackieviruses.

## Introduction

Coxsackievirus B3 (CVB3) is a member of the human enterovirus, which belongs to the *Picornaviridae* family. CVB3 has a single-stranded positive-sense RNA genome enclosed in a non-enveloped icosahedral capsid made of four capsid proteins (VP1 to VP4).[1–4] The spectrum clinical infections caused by CVB3 ranges from light to serious cases including acute and chronic inflammations. CVB3 may spread from the intestinal tract to internal organs and to cause acute heart failure and aseptic meningitis.[4–7]

The antiviral agent pleconaril is a potential drug for treatment of enteroviral infections in neonates or infants, but the experience with its use is still limited.[8] Ribavirin, a member of the nucleoside antimetabolite drugs that interfere with duplication of viral genetic material, is active against a number of DNA and RNA viruses, especially for Influenza, flaviviruses, and many pathogens that cause various viral haemorrhagic fevers. Moreover, one study reported that ribavirin (at a concentration of 11.59±5.47 μg/ml) inhibited the cytopathic effect (CPE) of CVB3.[9] According to Kishimoto et al. (1988), CVB3 infection in murine myocarditis has been treated with ribavirin which inhibits viral replication, resulting in a reduction in myocardial damage.[10,11] However, this antiviral agent is not a specific therapeutic option in the clinical practice used to treat CVB3 infections, [4] and there is no vaccine for CVB3. Therefore, novel strategies for control of CVB3 should be investigated.[9]

Over the past few decades, studies on the health-beneficial effects of probiotics or the substances that they naturally produce have actively been progressing in various fields. Probiotics are defined as microorganisms administered in adequate amounts that exert health-beneficial effects on the host.[12] These are mostly comprised of lactic acid-producing bacteria (LAB) such as *Lactobacillus* and *Bifidobacterium* species, which are significant gastrointestinal tract (GI) residents.[13] Probiotics are widely used and are organisms that are considered to be safe for applications in animals and humans.[14]

Probiotics support the functional and balanced state of the immune system in its role to combat microbial pathogens, such as viruses, and directly benefit the animal host by preventing infection or indirectly through restoration of the disrupted equilibrium of the enteric flora.[12,15] Several studies have demonstrated that probiotics enhance the antiviral effect against human rotaviruses, influenza virus and herpes viruses.[12,16,17]. Some probiotics inhibit the growth of viral pathogens by blocking binding sites on epithelial cells.[13] In addition, probiotics can diminish the risk for development of respiratory tract infections, which in most cases are of viral genesis.[18] However, to the best of our knowledge, the *in vitro* effect of probiotics against CVB3 has not been reported. The aim of this study was to examine *in vitro* the antiviral activity against CVB3 of cell extracts from probiotics isolated from human intestinal microflora. CVB3 replication induces a direct CPE on infected cells and direct tissue damage in various animal models.[4,19]. The plaque reduction assay has been accepted as the ‘gold standard’ for assessing the antiviral activity of materials against this particular virus.[9] Therefore, the antiviral activity of probiotic sample isolates against CVB3 was measured by the plaque reduction assay. We also investigated the inhibitory effects of probiotics against CVB3 using a real-time quantitative PCR (RT-qPCR). It is an excellent method for quantification of viral RNA.[20]

## Materials and methods

### Preparation of probiotic samples

Written informed consent was obtained from all volunteers who provided samples, and the protocol was approved by the Institution Review Board of the Office of Research Development, Sahmyook University.

Isolation of probiotic samples from healthy Koreans (20–30 years old) was done from their fecal specimens collected by BBL anaerobic sample collection and transport system (Becton Dickinson and Co., USA) to maintain anaerobic conditions. Fecal samples were diluted 10-fold from 10^−1^ to 10^−8^, and seeded onto selective blood liver agar (Nissui Pharm, Japan) containing 5% sheep blood. After 48 h incubation in anaerobic conditions (90% N_2_, 5% H_2_, 5% CO_2_) (Bactron Anaerobic Chamber, USA) at 37 °C, brown or reddish-brown colonies, 2–3 mm in diameter, were selected for further identification.[21] A fructose-6-phosphate phosphoketolase (F6PPK) test was performed to ensure that the selected colonies were probiotic samples.[22,23] To identify the isolated probiotic species, 16S rRNA sequencing was performed by Bio leaders (Daejeon, Korea) (Table 1). All probiotic samples were cultured at 37 °C for 48 h in selective general anaerobic medium (GAM, Nissui Pharm, Japan) under anaerobic conditions and then harvested for the preparation of probiotic cell extracts. Cultures were centrifuged at 4000 rpm for 10 min, washed with phosphate buffered saline (PBS), and resuspended in the same buffer. These bacterial suspensions were adjusted to a final concentration of 9.0 log colony forming unit (CFU)/ml and sonicated for 5 min (amplitude 100%) to lyse the cells. Cell extracts were filtered using 0.45-μm syringe filter (Sartorius Stedim Biotech, Germany) and used for further experiments.

**Table 1.** List of 13 probiotics isolated from human intestinal microflora.

	Origin			
Strains	Kind	Sex	Age	Result of BLAST search
*Bifidobacterium adolescentis*				
SPM 0212	Human	Female	21	99% identity with ATCC 15703
SPM 0214	Human	Female	21	99% identity with ATCC 15703
SPM 0308	Human	Female	22	99% identity with ATCC 15703
SPM 1005	Human	Male	25	99% identity with isolate L2-32
SPM 1604	Human	Male	20	99% identity with ATCC 15703
SPM 1605	Human	Male	20	99% identity with ATCC 15703
SPM 1608	Human	Male	20	99% identity with ATCC 15703
*Bifidobacterium pseudocatenuratum*				
SPM 1204	Human	Female	22	99% identity with JCM 1200
SPM 1309	Human	Male	24	99% identity with JCM 1200
*Bifidobacterium longum*				
SPM 1205	Human	Female	22	99% identity with DJO10A
SPM 1206	Human	Female	22	99% identity with DJO10A
SPM 1207	Human	Female	22	99% identity with DJO10A
*Lactobacillus ruminis*				
SPM 0211	Human	Female	21	99% identity with NBRC 102161

### Cells and viruses

Human cervical cancer HeLa cells were used for cultivation and titre confirmation of CVB3. The HeLa cells were cultured in Dulbecco's modified Eagle's medium (DMEM, Lonza, USA) containing 10% heat-inactivated fetal bovine serum (FBS, Sigma, USA), 1% (v/v) penicillin (10,000 units/ml) and streptomycin (10,000 units/ml) (Lonza, USA) at 37 °C with 5% CO_2_ atmosphere. For viral cultivation, the HeLa cells were seeded in T-75 flasks (NunC, Denmark) at a density of 1 × 10^7^ cells and incubated for 24 h. When cell confluency reached approximately 90%, cells were infected with CVB3 (MOI = 10) and incubated for 48 h under 5% CO_2_ atmosphere at 37 °C. The virus titres and probiotic samples were screened by plaque reduction assay.

### Cytotoxicity assay

The cytotoxicity of probiotic samples to the cultured cells was assessed by the MTT [3-(4,5-dimethylthiazol-2yl)-2,5-diphenyltetrazolium bromide] (Sigma Chemical Co., USA) assay. HeLa cells were seeded on 96-well microplates (NunC, Denmark) at a density of 1 × 10^4^ cells per well with 10% (v/v) probiotic samples and incubated for 24 h. The cells with culture media alone were used as a non-treated control. The MTT reagent (5 mg/ml in distilled water) was prepared immediately prior to use. The incubation medium of each well was removed, the cells were washed with PBS and 10 μl of MTT reagent was added. After incubation for 4 h at 37 °C, MTT reagent in 100 μl of dimethylsulfoxide (DMSO) was added to each well. Survivable cells were then detected by measuring absorbance at 570 nm with a plate reader. The cell viability was expressed as a percentage of the values that were obtained for the controls.

### Screening of probiotic samples by Plaque reduction assay

HeLa cells were seeded into six-well plates (6 × 10^5^ cells/well) and incubated at 37 °C with 5% CO_2_ for 48 h. When cell confluency reached approximately 90%, cells were washed with PBS to remove the fetal bovine serum and then overlaid with 200 μl of mixture containing the virus and the 10% (v/v) probiotic samples. As a control, HeLa cells were treated with same amount of mixture containing the virus and 10% (v/v) PBS. The cells were incubated at 37 °C with 5% CO_2_ for 60 min with rotation at every 10 min and the supernatants were removed. Finally, cells were overlaid with 1% SeaPlaque agar (Lonza, USA), an overlay consisting of 1x DMEM. The cells were incubated at 37 °C for 48 h, fixed with fixative (acetic acid: methanol at a ratio of 1:3) for 10 min and then stained with 1% crystal violet.

### Real-time quantitative PCR assay

HeLa cells were treated with the extracts and 200 μg/ml of ribavirin (Sigma, USA) as described above. For extracellular assays, viral RNA was extracted from culture supernatant with Ribospin vRD Kit (GeneAll, Korea) according to the manufacturer's recommendations. For the intracellular assay, total RNA from each of the cells was extracted with RNeasy® Mini Kit (Qiagen, Germany). Then, cDNA was synthesized by using Oligo-dT primer and Omniscript® Reverse Transcription kit (Qiagen, Germany). RT-qPCR was conducted in StepOnePlus™ Real-Time PCR (Applied Biosystems, USA) with a power SYBR Green PCR master mix (Applied Biosystems, UK), and data were analysed with StepOne™/StepOneplus™ Software version 2.0. The primer sequences used for RT-qPCR of CVB3-E1 and E2 were 5′-CCC CTG AAT GCG GCT AAT CC-3′ (nt 453-nt 472) and 5′-CAA TTG TCA CCA TAA GCA GCC A-3′ (nt 601-nt 580), respectively.[25] GAPDH was used as internal control with primer sequences of 5′-CCA TCA CCA TCT TCC AGG AG-3′ (forward) and 5′-CCT GCT TCA CCA CCT TCT TG-3′ (reverse). The amplification conditions were as follows: initial incubation at 95 °C for 10 min, followed by 40 cycles of 95 °C for 15 s, 60 °C for 1 min and 72 °C for 10 s. At least three independent assays were performed and positive and negative controls were included in each experiment to ensure reproducible results. Relative quantification of the target gene was calculated using the delta–delta CT (DDCT) method.[26]

### Statistical analysis

Results were expressed as mean ± standard deviation (SD). Significant differences were separated using Duncan's multiple range tests and commercial statistical analysis software, version 9.0 (SAS Institute, Cary, NC). All data were considered significant at *p* < 0.05.

## Results and discussion

### Cytotoxicity assay

**Figure 1.**
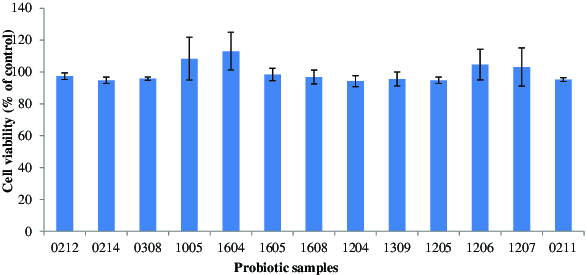
Cytotoxicity of probiotics in HeLa cells. Experiments were performed by means of an MTT enzyme assay. HeLa cells were incubated in the presence of cell extracts from various probiotic samples at 37 °C for 24h. Each column represents the mean ± SD with respect to 100% control. At least three independent assays were performed.

The results from MTT assay showed that all bifidobacteria samples were not cytotoxic to HeLa cells (Figure 1).

### Screening of probiotic samples by plaque reduction assay

**Figure 2.**
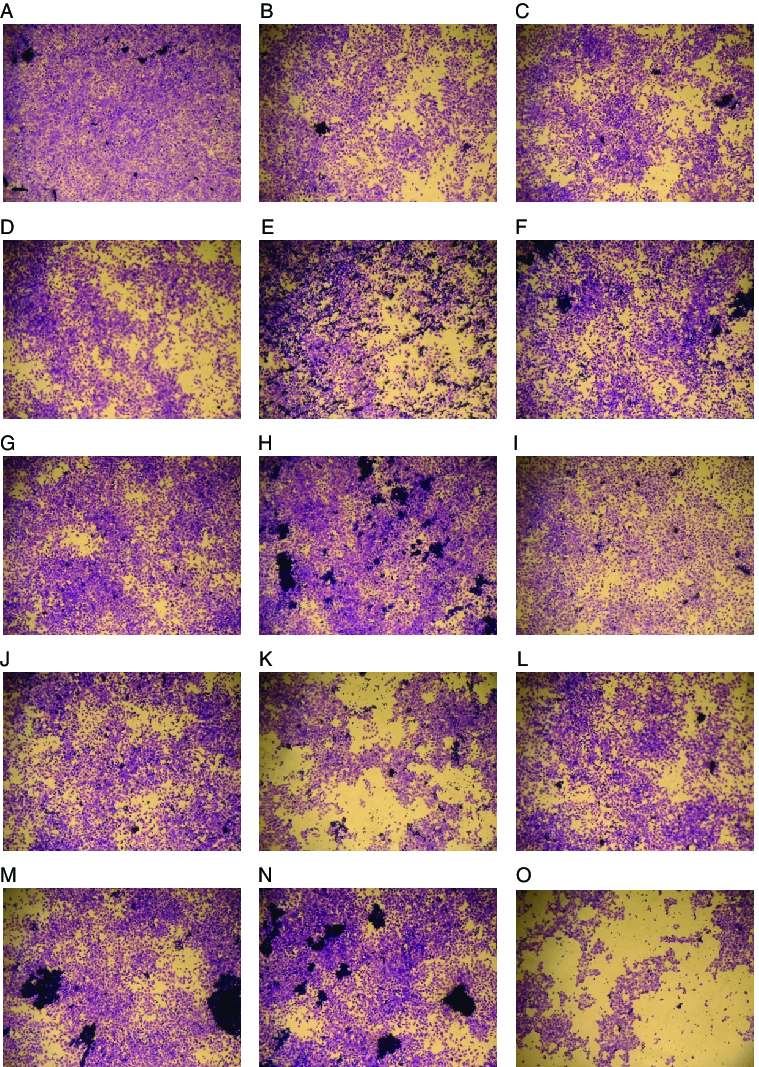
Antiviral activity of 13 probiotic samples against CVB3. HeLa cells were treated with cell extracts from various probiotic samples and the plaque reduction assay was conducted. HeLa cells were observed under a light microscope at ×200. (A) No infection; (B) CVB3 infection; (C) CVB3+SPM0212; (D) CVB3+SPM0214; (E) CVB3+SPM0308; (F) CVB3+SPM1005; (G) CVB3+SPM1604; (H) CVB3+SPM1605; (I) CVB3+SPM1608; (J) CVB3+SPM1204; (K) CVB3+SPM1309; (L) CVB3+SPM1205; (M) CVB3+SPM1206; (N) CVB3+SPM1207; (O) CVB3+SPM0211. At least three independent assays were performed.

We confirmed the antiviral activity of 13 probiotic samples against CVB3 by using plaque reduction assay. Three *B. adolescents* (SPM1005, 1604 and 1605), two *B. longum* (SPM1206 and 1207) and one *B. pseudocatenulatum* SPM1204 samples had an inhibitory effect against CVB3, and the rest did not show such inhibition. Although some probiotic samples contained the same species, their effects were different (Figure 2). Among all tested bifidobacteria samples, *Bifidobacterium adolescentis* SPM1605 showed the greatest antiviral activity (Figure 2(H)). *B. adolescentis* SPM1605 and *B. adolescentis* SPM1608 are different strains from the same species, but *B. adolescentis* SPM1608 did not demonstrate an inhibitory effect (Figure 2(H) and 2(I)). Therefore, this effect was not species-specific but rather strain-specific.

### Inhibitory effect of *B. adolescentis* SPM1605 on CVB3

**Figure 3.**
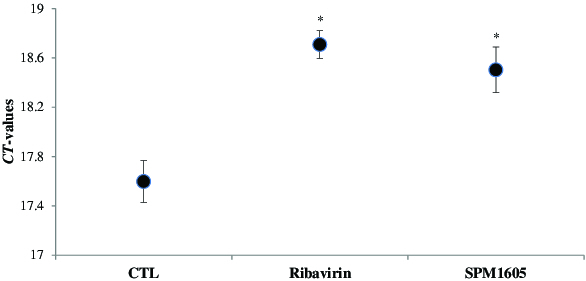
Comparison of CT values between CVB3-infected-HeLa cells treated with *B. adolescentis* SPM1605 and non-treated cells. CVB3 infected-HeLa cells were treated with the cell extract of *B. adolescentis* SPM1605 at 9.0 log CFU/ml, and the levels of extracellular CVB3 RNA were analysed by real-time qPCR. The used concentration of ribavirin was 200 μg/ml. At the threshold cycle (CT), a detectable amount of amplification product has been generated during the early exponential phase of the reaction. The threshold cycle is inversely proportional to the original relative expression level of the gene of interest. At least three independent assays were performed. *p < 0.05 significantly different compared with control (CTL).

**Figure 4.**
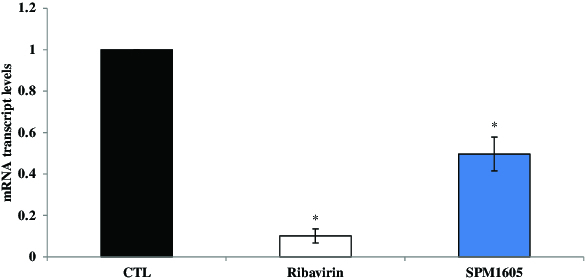
Effects of *B. adolescentis* SPM1605 cell extract on CVB3 replication. CVB3-infected-HeLa cells were treated with the cell extract of *B. adolescentis* SPM1605 at 9.0 log CFU/ml, and the levels of intracellular CVB3 RNA were analysed by real-time qPCR. The used concentration of ribavirin was 200 μg/ml. At least three independent assays were performed. *p < 0.05 significantly different compared with control (CTL).

To determine the effect from the cell extract of *B. adolescentis* SPM1605 on the replication of CVB3, we performed an RT-qPCR analysis using specific primers for a CVB3 gene. The RT-qPCR results showed that replication of CVB3 was significantly inhibited by *B. adolescentis* SPM1605, although it was less efficient than the application of ribavirin (200 μg/ml). When the critical threshold (CT) values between the treated *B. adolescentis* SPM1605 sample and non-treated ones were compared, the extracellular level of CVB3 RNA was shown to be significantly reduced by *B. adolescentis* SPM1605 (Figure 3). Moreover, the intracellular level of CVB3 RNA was also inhibited by 50% (Figure 4).

Probiotics, particularly bifidobacteria, are used in yogurt and other fermented milk products and are widely assumed to be safe. Probiotics have demonstrated many beneficial effects on human health such as in the intestinal tract for the treatment of gastrointestinal diseases; and as contribution to the immune modulatory effects in combatting microbial pathogens, including viruses.[26] Probiotics may affect the replication of human enteric viruses involved in non-enteric pathologies. Several clinical studies indicate that probiotics can shorten the duration of viral diarrhoea. Children that received bifidobacteria-supplemented formula had a reduced risk for development of diarrhoea and a rotaviral infection.[27] Some *Lactobacillus* strains reduce the period of rotavirus excretion. In an European multicentre trial, oral administration of *Lactobacillus* GG reduced the duration of disease in children with acute diarrhoea.[28,29]

Furthermore, *B. adolescentis* SPM0214 inhibited the replication of Herpes simplex virus-1 (HSV-1) by 30%.[18] The cell extract of *B. adolescentis* SPM0212 decreased the extracellular HBV surface antigen (HBsAg) levels by up to 50% and the mRNA transcript level of HBsAg in HepG2.2.15 cells by 40%.[30] In addition, *Lactobacillus* strains were used to prevent and treat infection with influenza virus and for protection against other respiratory infections.[17]

In this study, some probiotics, such as three *B. adolescents* (SPM1005, 1604 and 1605), two *B. longum* (SPM1206 and 1207) and one *B. pseudocatenulatum* SPM1204 samples, were determined to exhibit antiviral activity against CVB3. Among them, *B. adolescentis* SPM1605 showed the greatest antiviral activity. Several studies showed that effects of probiotics were not species-specific but rather strain-specific.[18,30] Likewise, antiviral activity of *B. adolescentis* SPM1605 was strain-specific. This strain significantly inhibited the extracellular and intracellular levels of CVB3 RNA by approximately 50%. However, the mechanism of antiviral activity of *B. adolescentis* SPM1605 was not obviously explained. Several mechanisms have been suggested for the antiviral activity of probiotics, inhibiting the adsorption of viral particles or blocking their entry into cells,[13,14] modulation of signal transduction pathways involved in any step of the viral cycle, such as those used by CVB3 for viral entry (Abl kinase),[13] and altering the state of the intestinal cells and macrophages, leading to an antiviral response.[14]

According to one study, the antiviral mechanism of *B. adolescentis* SPM0212 cell extract involved the pathways of the IFN-mediated antiviral response. The IFN family of cytokines is now recognized as a key component of the innate immune response and as the first line of defence against viral infections. Gene targeting research has identified four essential effector pathways of the IFN-mediated antiviral response: the Mx GTPase pathway, the 20,50-oligoadenylate-synthetase-directed ribonuclease L pathway, the PKR pathway and the ISG15 ubiquitin-like pathway. These effector pathways individually block viral transcription, inhibit translation, degrade viral RNA and convert protein function to control all steps of viral replication.[30,31] Gene expression of IFN-signalling components was increased by *B. adolescentis* SPM0212 cell extract. *B. adolescentis* SPM0212 cell extract also increased MxA gene expression.[30] MxA protein binds viral nucleocapsids or other viral components and then degrades them. Viruses that are susceptible to the activities of MxA include hepatitis B virus, orthobunyavirus, hanta virus, phlebovirus, dugbe virus as well as coxsackieviruses. Moreover, the antiviral activity of MxA proteins against CVB has been studied [32] and it may be suggested that *B. adolescentis* SPM1605 may have antiviral activity by affecting the MxA gene expression.

## Conclusions

*B. adolescentis* SPM1605 demonstrated antiviral activity against CVB3. Moreover, as *B. adolescentis* SPM1605 is considered safer than other compounds, it may be ingested with food and medicine. We therefore suggest that *B. adolescentis* SPM1605 may be an alternative therapy for infection with coxsackievirus. Despite various reports that indicate a protective effect of probiotics in epidemiological studies, the antiviral effects have not been studied in details yet. Thus, the mechanism of action in relation to the antiviral effects needs to be further investigated regarding the properties of probiotics in various clinical conditions.
